# Editorial policies of Journal of Educational Evaluation for Health Professions on the use of generative artificial intelligence in article writing and peer review

**DOI:** 10.3352/jeehp.2023.20.40

**Published:** 2023-12-31

**Authors:** Sun Huh

**Affiliations:** Department of Parasitology, Institute of Medical Education, College of Medicine, Hallym University, Chuncheon, Korea; Hallym University, Korea

## Background

After the public was introduced to the generative artificial intelligence (AI) platform known as ChatGPT on November 30, 2022 [1], researchers began to use generative AI, including AI chatbots, when writing articles [2].

Generative AI is a type of AI technology capable of generating text, images, or other media based on the data on which it has been trained [3]. An AI chatbot is a specific application of generative AI designed to simulate conversations with human users [4]. GPT stands for “generative pre-trained transformer”—an AI model designed for understanding and generating natural language [5]. Natural language refers to any language that occurs naturally in human communication, such as English or Korean [6]. Sign language is also treated as a natural language. An AI tool is a software application that uses AI algorithms to perform specific tasks and solve problems. It is a broader term than generative AI. Generative AI platforms are used interchangeably with “generative AI,” but platforms are defined as more specific places or sites on the web that use generative AI technologies. Examples of platforms include ChatGPT (https://chat.openai.com/), Bard (https://bard.google.com/), Bing (https://www.bing.com/), or Clova X (https://clova-x.naver.com/).

In this editorial, “AI chatbot” is used with the same meaning as “generative AI,” although “generative AI” is a broader term. The term “AI tool” is also confined to the sense of generative AI in the issues of article writing and review. “Generative AI platforms” are also used synonymously with “chatbots.”

Although there have been many AI-assisted tools for article writing [7], generative AI provides unimaginable research tools. In particular, non-native English researchers use these tools for translation or English proofreading. The scientific community began to discuss the use of ChatGPT, a generative AI platform. Avi Staiman, MA Founder & CEO of Academic Language Experts, presented thoughtful opinions on using the generative AI platform at the 2023 Council of Science Editors Annual Meeting on May 2, 2023. He asked, “Why do we have such an emotional reaction to AI and ChatGPT? AI has been around for a long time, but ChatGPT is different because it has the power to displace information workers and impact our knowledge economy.” He discussed the clients of AI platforms in the research area—non-native English-speaking authors, cooperative research advisors, research assistants, personal peer reviewers, and personal publicists for media engagement [8]. His stance on generative AI aligns with my opinion.

I have sometimes received manuscripts that are generated by AI platforms. I have also checked the manuscript for duplicate publication with Crosscheck (Similarity Check). Besides this well-known editors’ tool, tools exist for checking whether texts have been generated by AI, such as ZeroGPT (https://www.zerogpt.com) or GPTZero (https://gptzero.me). I have frequently used generative AI platforms for translation, paraphrasing, grammar checking, English proofreading, information flow checking, statistical analysis, abstracting, figure generation, reference seeking, reference style adjustment, and research topic generation. Therefore, the use of generative AI is part of the everyday mandatory work of an author and editor. I have not used it for peer review of submitted manuscripts because when I tested AI platforms for peer review of my manuscript, the results were unsatisfactory. AI platforms are sometimes used to improve the presentation when I edit manuscripts. However, they are not used for publication because publication is done by a publishing company. AI platforms cannot yet generate a PDF or XML from a word processor file.

I have sometimes been asked to suggest guidelines on using generative AIs for editors. The *Korean Journal of Radiology* is a pioneer journal in this area [9], and it will serve as a useful reference for academic society journal editors.

## Objectives

This editorial presents policies on using generative AI platforms (AI chatbots) for writing and reviewing manuscripts in the *Journal of Educational Evaluation for Health Professions* (JEEHP). These policies may be changed according to the development of generative AI platforms and transitions in the publishing environment. Furthermore, the next editor of the journal can revise the guidelines. My suggestions are solely based on my experience as an editor, reviewer, and author.

## Data sources/analysis

Literature and guidelines on this topic are gathered and analyzed. After that, the editor’s opinion is suggested.

## Comparison of guidelines of editors’ organizations and academic journals

There are some guidelines on the use of generative AI platforms. Table 1 summarizes the guidelines from international editors’ organizations. There was no clear guideline on this topic from the European Association of Science Editors or the Council of Asian Science Editors. Therefore, those from the Council of Science Editors [10], the Committee of Publication Ethics [11], the World Association of Medical Editors [12], and the International Committee of Medical Journal Editors [13] were included. As a guideline from academic society journals, those from the *Korean Journal of Radiology* [9] were added, since those policies were well-designed. In the last column, the guidelines of JEEHP were also added. These guidelines are described in more detail in the next section.

## Guidelines on the use of generative AI in writing and review for the *Journal of Educational Evaluation for Health Professions*

### Generative AI as an author

All organizations forbid generative AI from being listed as an author. There are reasons as follows: an author must be a legal person; chatbots do not meet the International Committee of Medical Journal Editors (ICMJE) authorship criteria; they cannot approve the final version; and they cannot be accountable for all aspects of the work, including disclosure of a conflict-of-interest statement [13]. In a previous editorial, I also stated that generative AI could not be an author [2]. Institutions are already frequently listed as authors, although they are not human. At this time, listing an institution as the author conveys the meaning of referring to all “human” authors affiliated with an institution. Even if the author is listed as an institution, the principle of human authorship is still maintained. Editors usually do not require the institutes’ ORCID, conflict of interest disclosure, or approval of the final version.

There is a case of a textbook written by an AI entity, “Beta Writer,” on lithium-ion batteries. This book’s intrinsic value is anchored in its origination from an AI, heralding a new era of scientific discourse and fostering subsequent research advancements. However, this work was led by its project directors. Generative AI is different from the case of “Beta Writer” because generative AI does not meet the required qualifications for accountability [14].

Journals such as *Nature* have said that generative AI platforms (AI chatbots) cannot be listed as authors in their articles. According to their rules about who can be an author, AI technologies do not yet meet the necessary standards, especially in being responsible for what they write [15]. Generative AI platforms also cannot fulfill ICMJE’s 4 conditions of authorship. As for JEEHP, my stance is that I recognize generative AI platforms as tools, analogous to statistical packages. Therefore, I do not want to allow the authorship of generative AI.

### Authors’ disclosure of the use of AI tools

All organizations have mandated this item. However, the problem is that it is not easy to detect it. Although there are tools to detect AI-generated text, they have limitations. The results of each tool are sometimes different. Second, the boundary between allowable generation for assistance and not-allowable primary generative writing is unclear. Third, it is common for authors to use generative AI platforms for writing and editing articles, so that the primary generated texts and the author’s revision may be mixed. For these reasons, I may reconsider the necessity of mandatorily reporting AI-generated content to the editor. Furthermore, the disclosure of AI use to peer reviewers and editors is optional, not mandatory. The well-known plagiarism detection programs, such as Similarity Check (Crosscheck, https://www.crossref.org/services/similarity-check/) and CopyKiller (https://www.copykiller.com/), are unable to screen for sentences and text produced by generative AI [16]. Plagiarism checking for AI-generated text is the editor’s responsibility.

### Asking authors to verify the use of AI tools

From the same viewpoint as the previous statement, asking authors to verify the use of AI tools is not required. If the authors want to verify it, they can do so at their discretion. The decision depends on the author’s choice. Statistical analysis can now be performed by generative AI platforms. In such cases, authors can incorporate generative AI for statistical programs instead of relying solely on well-known statistical packages. It would be natural to cite generative AIs in this case.

### Journal’s explicit policy about the use of AI-generated text and images

If AI tools are thought to be statistical tools or writing tools, it is not required for a journal to disclose explicit policy about the use of AI-generated text and images. The editors can select items in Table 1 and modify them accordingly. Therefore, this policy can be optional for editors. However, JEEHP has announced its policies about the use of AI-generated text and images in this editorial.

### Author’s accountability (responsibility)

The author’s accountability is always emphasized in all writing, not only AI-generated writing. All content of the manuscripts or published articles should be under the author’s responsibility. OpenAI announced ownership of content as follows: “As between you and OpenAI, and to the extent permitted by applicable law, you (1) retain your ownership rights in Input and (2) own the Output. We hereby assign to you all our right, title, and interest, if any, in and to Output” [17]. This means that the output generated by the user is owned by the user and OpenAI does not have responsibility for the output. However, science continuously evolves, and a disclaimer on the content is necessary for each journal.

### Editors’ and reviewers’ specification of the use of chatbots in review and correspondence

As authors’ disclosure of the use of generative AI is optional, editors and reviewers do not need to specify this information.

### Reviewers uploading manuscripts to AI tools where confidentiality is not assured

If uploaded manuscripts are not secure, they should not be uploaded for peer review. The problem is that if reviewers upload a manuscript to an AI platform, there is no way for editors to detect it. Therefore, it depends on the reviewer’s choice whether they adhere to this recommendation or not. Furthermore, it is uncertain which generative AIs use uploaded manuscripts for their pre-training for performance improvement. For example, OpenAI announced that “data submitted through non-API [application programming interface] consumer services ChatGPT or DALL•E may be used to improve the models,” and that users of that platform can request to opt out of having their data used [18]. If there is a lucid statement that a platform does not use uploaded data for pre-training, uploading a manuscript for peer review will be no problem.

### Availability and use of a tool to check for AI-generated text

There are many tools for this purpose. The editorial office has already used some of them, as previously mentioned.

### Use of generative AIs to enhance linguistic quality

This is unnecessary to mention because it is already routine work for authors.

### Research on generative AI

A clear statement regarding AI use for research on AI is natural. It should be mentioned in the Methods section.

### AI-generated text or output as a supplement and citing AI-generated text in the main text

The data generated by AI cannot be reproduced because generative AI systems evolve rapidly, so the answers vary over time. Therefore, using AI-generated data, the answers can be presented as supplemental materials. No consensus exists on listing the generative AI product as a supplement. This was already suggested in the previous editorial, as follows [2]: Example> Supplement. Answer of GPT-4 to the inquiry, “What is the definition of generative artificial intelligence?” (cited 2023 Dec 25–27 [KST]).

It is not meaningful to cite AI-generated text or images in the References section because they are not reproducible with the same prompt. To cite AI-generated texts, the texts should be added as a supplement.

### Generative AI listed in the Acknowledgments section

I acknowledge generative AI as a research tool. As with authorship, I do not allow authors to list generative AI platforms in the Acknowledgment section.

## Limitations

These policies reflect the opinions of this journal editor. They may be changed according to changes in the performance of generative AI, and a new editor or publisher could establish different policies.

## Generalizability

Although these are the policies and guidelines for a single journal, the content can be used selectively for other scholarly journals.

## Conclusion

The inevitability of authors utilizing generative AI, such as AI chatbots, for article writing is now undeniable. If these tools can enhance data analysis and interpretation, thereby aiding in article composition, it becomes imperative to encourage researchers to use these tools to reduce time and costs. This editorial presents guidelines for the more active and effective use of generative AI by authors. These guidelines may differ from those of various organizations, but they might be adequate if chosen and applied by editors of other academic journals. Many tasks involved in article writing, including statistical analysis and the creation of figures and tables, can now be carried out using AI. Consequently, researchers equipped with data can expedite the writing process. However, it is advisable to limit the extent of AI-generated text and ensure that the author’s original thoughts and creativity are prominently featured in the article.

## Figures and Tables

**Table 1.** Comparison of guidelines on the use of generative artificial intelligence from international organizations and academic society journals

Items	CSE	COPE	WAME	ICMJE	KJR	JEEHP
Date of announcement	June 2023	February 13, 2023	May 31, 202	May 20123	August 2023	December 2023
1. Authorship	Not allowed	Not allowed	Not allowed	Not allowed	Not allowed	Not allowed
2. Authors’ disclosure of the use of AI tools	Required	Required	Required	Required	Required	Optional
3. Asking authors to discloses the use of AI tools	Recommend	-	Required	Required		No
4. Journal’s explicit policy about the use of AI-generated text and images	Required	-	Required	-	Present	Present
5. Author’s accountability (responsibility)	Yes	Yes	Yes	Yes	Yes	Yes
6. Editors’ and reviewers’ specification of the use of chatbots in reviews and correspondence	-	-	Required	Required	-	Optional
7. Reviewers uploading manuscripts to AI tools where confidentiality is not assured.	-	-	-	Not allowed	Not allowed	Not allowed
8. Availability and use of a tool to check for AI-generated text	Will be announced	-	Needed	-	-	Being used
9. Use of AI to enhance linguistic quality	-	-	-	-	Acceptable; no disclosure required	Acceptable
10. Research on AI	-	-	-	-	Details of the use of AI tools should be disclosed	Same as other articles

CSE, Council of Science Editors; COPE, Committee of Publication Ethics; WAME, World Association of Medical Editors; ICMJE, International Committee of Medical Journal Editors; KJR, *Korean Journal of Radiology*; JEEHP, *Journal of Educational Evaluation for Health Professions*; AI, artificial intelligence.
